# Exploring the Ligand Efficacy of Cannabinoid Receptor 1 (CB1) using Molecular Dynamics Simulations

**DOI:** 10.1038/s41598-018-31749-z

**Published:** 2018-09-13

**Authors:** Sang Won Jung, Art E. Cho, Wookyung Yu

**Affiliations:** 1 Center for Supercomputing and Big Data, DGIST, 333 Techno jungang-daero, Daegu, 42988 Korea; 2 Department of Bioinformatics, Korea University, 2511 Sejong-ro, Sejong, 30019 Korea; 3 Department of Brain and Cognitive Sciences, DGIST, 333 Techno jungang-daero, Daegu, 42988 Korea; 4 Core Protein Resources Center, DGIST, 333 Techno jungang-daero, Daegu, 42988 Korea

## Abstract

Cannabinoid receptor 1 (CB1) is a promising therapeutic target for a variety of disorders. Distinct efficacy profiles showed different therapeutic effects on CB1 dependent on three classes of ligands: agonists, antagonists, and inverse agonists. To discriminate the distinct efficacy profiles of the ligands, we carried out molecular dynamics (MD) simulations to identify the dynamic behaviors of inactive and active conformations of CB1 structures with the ligands. In addition, the molecular mechanics Poisson-Boltzmann surface area (MM-PBSA) method was applied to analyze the binding free energy decompositions of the CB1-ligand complexes. With these two methods, we found the possibility that the three classes of ligands can be discriminated. Our findings shed light on the understanding of different efficacy profiles of ligands by analyzing the structural behaviors of intact CB1 structures and the binding energies of ligands, thereby yielding insights that are useful for the design of new potent CB1 drugs.

## Introduction

Cannabinoid receptors are class A members of the G-protein coupled receptor (GPCR) superfamily^1^. Two subtypes of cannabinoid receptors are currently known: cannabinoid receptor 1 (CB1)^2,3^, which is located in the brain and many peripheral organs and tissues, and cannabinoid receptor 2 (CB2)^4^, which is mainly expressed in immune cells. CB1 is the most abundant GPCR in the brain and central nervous system that regulates a variety of brain functions and behaviors such as pain, control of movement, memory, and neuroendocrine regulation^5–7^. In addition, CB1 in peripheral organs and tissues has been shown to play an important role in physiological mechanisms such as energy metabolism, appetite control, endocrine, and metabolic regulation^8,9^. Thus, CB1 is a promising therapeutic target for a variety of disorders.

Depending on the biological response of CB1, ligands with different efficacy profiles and therapeutic effects were largely classified into three classes: agonists, antagonists, and inverse agonists^10–12^. CB1 agonists have potential for therapeutic applications in pain, inflammation, and neurodegenerative disorders^13,14^. The Δ^9^-tetrahydrocannabinol (THC)^15^, which is the psychoactive constituent of marijuana, is known as a CB1 partial agonist and is used for therapeutic purposes such as analgesic, antiemetic, and anticonvulsant in the USA and other countries^16,17^. Meanwhile, CB1 antagonists and inverse agonists have been developed for therapeutic applications in obesity-related metabolic disorders, mental illness, liver fibrosis, and nicotine addiction^18–20^. The Δ^9^-tetrahydrocannabivarin (THCV)^21^ is a CB1 antagonist that is structurally similar to THC. However, unlike THC, THCV has anti-obesity activity. CB1 inverse agonists such as rimonabant^22^ and taranabant^23^ are also effective in treatment of obesity, but psychiatric side effects such as anxiety and depression have been reported^24,25^. Therefore, with regard to the distinct efficacy of the three classes of ligands, sophisticated drug design strategies are required to achieve the desired therapeutic effects.

Recently, two conformations of CB1 crystal structures have been determined: (1) the inactive conformation bound to the antagonist AM6538^26^ or inverse agonist taranabant^27^; and (2) the active conformation bound to the agonist AM11542 or AM841^28^. There were significant structural changes between the two conformations, especially in helices I, II, and VI. The extracellular part of helices I and II move inwards, and the intracellular part of helix VI moves outwards, thereby shrinking the volume of the orthosteric ligand-binding site by 53%^28^. In addition, the conformational changes of a twin toggle switch of Phe200 and Trp356 were also observed. These findings provide new insights into the mechanisms of structural changes depending on two classes of ligands and how they are bound in the orthosteric ligand-binding site. Although the crystal structures of CB1 have been determined, a large amount of work still needs to be done in order to understand the dynamic behaviors of the two conformations of CB1 as well as to be able to design chemically diverse ligands with distinct efficacy profiles.

Here, we demonstrate the dynamic behaviors of intact CB1 structures when the three classes of ligands were bound to the active and inactive conformations for the discrimination of ligand efficacy through molecular dynamics (MD) simulations. One study by West *et al*. experimentally demonstrated that the distinct conformational states of beta-2-adrenergic receptor (β_2_AR) were induced by the diverse classes of ligands^29^. When the ligands were bound to the β_2_AR, the intra- and extracellular regions were notably changed and showed distinct conformations depending on the ligands. Thus, to investigate the dynamic behaviors of CB1 structure, the active and inactive conformations of CB1 structures were used to identify the structural rearrangement induced by ligand binding. Three classes of ligands, including THC as a partial agonist, THCV as an antagonist, and taranabant as an inverse agonist, were docked to the two structures. With these complex structures, MD simulations were carried out. In addition, the molecular mechanics Poisson-Boltzmann surface area (MM-PBSA) method was used to examine the binding energies of the three classes of ligands in the two conformations of CB1 and to determine the residual contributions of ligand binding. Our findings will help to discriminate the distinct efficacy profiles of the ligands and to provide new opportunities for the design of new CB1 drugs.

## Computational Methods

### Protein preparation

The crystal structures for the inactive and active conformations of CB1 (PDB ID: 5TGZ^26^ and 5XRA^28^) were obtained from the Protein Data Bank (PDB)^30,31^. Both structures were modified by mutating residues and inserting flavodoxin into the ICL3 region to facilitate crystallization. In order to perform molecular dynamics simulations, inactive and active conformations of wild-type intact CB1 structures were generated by reverting the mutant residues to wild-type and by reconstructing the ICL3 region using Modeller v9.18^32,33^, which was used in several studies for modelling ICL3 region in GPCRs^34,35^. A total of 20 structures for each conformation of CB1 were generated, and the one with the lowest discrete optimized protein energy (DOPE) score was selected. A loop refinement step was then performed to generate the 10 different loop structures, and the one adopted unstructured conformation with the lowest DOPE score was finally selected.

The two final structures, including the active and inactive conformations of the wild-type intact CB1 structures, were prepared using the protein preparation wizard^36,37^ module of the Schrödinger suite. The protonation and tautomeric states of Asp, Glu, Arg, Lys, and His residues were adjusted to match a pH of 7.0. The possible orientations of the Asn and Gln residues were generated. Finally, restrained minimization with the OPLS_2005 force field^38^ was performed with the hydrogens only option to optimize the hydrogen atom positions.

### Ligand preparation

All three ligand structures for Δ^9^-tetrahydrocannabinol (THC), Δ^9^-tetrahydrocannabivarin (THCV), and taranabant were initially drawn using the 2D-Sketcher and prepared using the LigPrep^39^ module of Schrödinger suite with the OPLS_2005 force field. LigPrep generated tautomers and stereoisomers within a pH range of 7.0 ± 2.0 using Epik^40–42^. Only the lowest energy conformer was retained for each ligand. Next, the ligands were optimized using the Jaguar^43,44^ module of the Schrödinger suite at the B3LYP/6–31 G* basis set. The calculated electrostatic potential (ESP) charges were used as partial charges for the ligands.

### Molecular docking

The three ligands were docked to the orthosteric binding site of the inactive conformation of the CB1 structure while two ligands, THC and THCV, were docked to the same site of the active conformation of the CB1 structure by using the Glide^45–47^ module of Schrödinger suite. Glide uses grids for fast scoring; the grid-generation module was used to generate grids for the two conformations of the CB1 structures. The van der Waals (vdW) scaling and partial charge cutoff was set to 0.8 and 0.15, respectively. Next, the SP mode of Glide was used to produce 5 poses per ligand, and the one pose with the lowest docking score was selected. In addition, the induced-fit docking (IFD)^48,49^ module of Schrödinger suite was used to dock the taranabant to the active conformation of the CB1 structure using default parameters, and the one pose with the lowest docking score was selected.

### System setup

A total of eight structures were used for the simulations: six CB1-ligand complex structures from the docking simulations as well as the inactive and active apo CB1 structures. The orientation of the CB1 structures with respect to the membrane were determined by using the Positioning of Proteins in Membrane (PPM) server of the Orientations of Proteins and Membranes (OPM) database^50^. The oriented proteins were inserted in the 1-palmitoyl-2-oleoyl-sn-glycero-3-phosphocholine (POPC) lipid bilayer using the CHARMM-GUI Membrane Builder^51–53^. The protein-membrane system was solvated with TIP3P^54^ water and 0.15 M NaCl. The final system size was approximately 79 Å × 79 Å × 111 Å in the inactive conformation and 88 Å × 88 Å × 116 Å in the active conformation. The force field parameters for the ligands were obtained using ParamChem^55,56^ with CHARMM general Force Field (CGenFF)^57^.

### Molecular dynamics simulations

All simulations were performed using GROMACS v5.1.4^58,59^ with the CHARMM36 force field^60,61^ for all compositions. Newton’s equations of motion were integrated using the leapfrog algorithm^62^. A 2 fs integration time step was used, with the bonds between hydrogen atoms and any heavy atoms constrained to their equilibrium lengths using the LINCS algorithm^63^. Periodic boundary conditions were used. For both vdW and electrostatic interactions, cutoffs of 1.2 nm were applied. Long-range electrostatic interactions were calculated using the particle mesh Ewald (PME) method^64^. The temperature was maintained at 310 K using a Nosé-Hoover thermostat^65,66^, with a coupling time constant of 1.0 ps. The system box was allowed to fluctuate under 1 atm using a semi-isotropic Parrinello-Rahman barostat^67^. All systems were minimized and then equilibrated for a total of 10 ns, including NVT and NPT with the Berendsen weak coupling method^68^. The z coordinates of the lipid atoms were restrained during the equilibration steps to restrict their motion to the x-y plane. After equilibration, the simulations were carried out for 1 μs under the NPT ensemble without any position restraints.

All trajectory analyses were performed by the analysis tools in GROMACS v5.1.4 package and VMD^69^. Root-mean-square deviation (RMSD) and root-mean-square fluctuation (RMSF) calculations, and distance evolutions were produced by GROMACS analysis tools.

### MM-PBSA binding energy calculations

In order to calculate the binding free energy of each protein-ligand complex, the MM-PBSA method was carried out using the g_mmpbsa^70^ tool. In total, 100 snapshots were extracted from the last 25 ns trajectory of each MD simulations. To get better statistics of binding free energy analysis, another set of MD simulations carried out. The binding free energy of each complex was computed by the following equation:

1 $${{\rm{\Delta }}{\rm{G}}}_{{\rm{bind}}}={{\rm{G}}}_{{\rm{complex}}}{-({\rm{G}}}_{{\rm{protein}}}+{{\rm{G}}}_{{\rm{ligand}}})$$

where G_complex_ indicates the free energy of the protein-ligand complex, and G_protein_ and G_ligand_ are the free energies of isolated protein and ligand in solvent, respectively. When calculating the free energy, G, the entropy contribution of the protein, was ignored because the binding energy was used here to determine the relative binding strength of each complex.

2 $${{\rm{G}}= < {\rm{E}}}_{{\rm{MM}}}{ > + < {\rm{G}}}_{{\rm{sol}}} > $$

where < E_MM_ > is the average molecular mechanics (MM) potential energy while using a CHARMM36 force field in a vacuum, and < G_sol_ > is the average solvation free energy.

The potential energy, E_MM_, was composed of two terms.

3 $${{\rm{E}}}_{{\rm{MM}}}{={\rm{E}}}_{{\rm{bonded}}}+{{\rm{E}}}_{\mathrm{non} \mbox{-} \mathrm{bonded}}$$

where E_MM_ = E_bond_ + E_angle_ + E_torsion_ and E_non-bonded_ = E_vdW_ + E_electrostatic_

The solvation free energy, G_sol_, was composed of two terms.

4 $${{\rm{G}}}_{{\rm{sol}}}{={\rm{G}}}_{{\rm{ps}}}{+{\rm{G}}}_{{\rm{nps}}}$$

G_ps_ was the polar solvation contribution calculated by solving the nonlinear Poison-Boltzmann (PB) equation. The values for the solute embedded in membrane (pdie) and solvent (sdie) dielectric constants were chosen to be 2 and 80, respectively. The nonpolar solvation free energy, G_nps_, was estimated by the solvent-accessible surface area using a water probe radius of 1.4 Å.

5 $${{\rm{G}}}_{{\rm{nps}}}={\rm{\gamma }}\mathrm{SASA}+b$$

where the constants γ and *b* were set to 0.00226778 kcal/mol·Å^2^ and 3.84928 kcal/mol, respectively.

In addition, per-residue free energy decomposition was performed to identify the contribution of individual residues to the binding free energy of the CB1-ligand complex.

## Results and Discussion

### Binding poses of ligands

**Figure 1:**
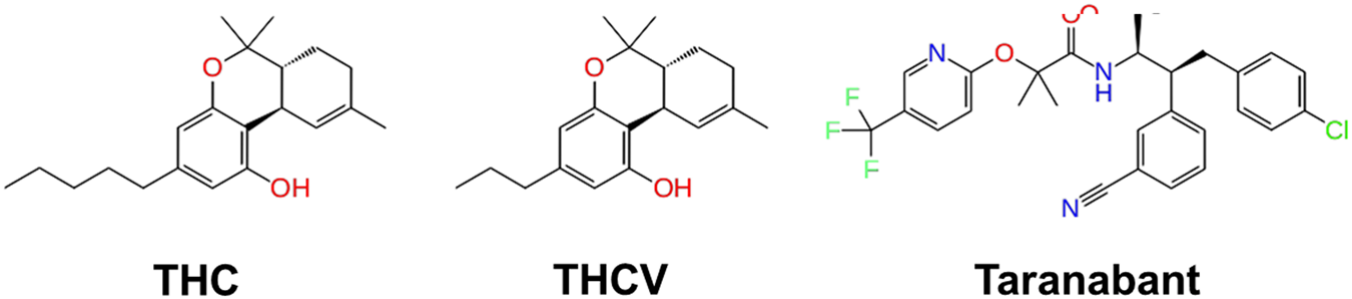
Chemical structure of the partial agonist THC, antagonist THCV, and inverse agonist Taranabant.

In this study, three classes of CB1 ligands were selected for exploring the ligand efficacy: THC as a partial agonist, THCV as an antagonist, and taranabant as an inverse agonist (Fig. 1). The partial agonist THC is one of the main psychoactive compound and is known to bind and activate CB1; thus, it is important to understand the binding mode of this ligand in the CB1 structure. The antagonist THCV is a propyl analogue of THC, but the effect of ligand efficacy on CB1 is different. It was interesting to examine how the CB1 structure was differently influenced by two structurally similar ligands. Taranabant is a potent CB1 inverse agonist, and the binding mode and its structural influence were also examined.

**Figure 2:**
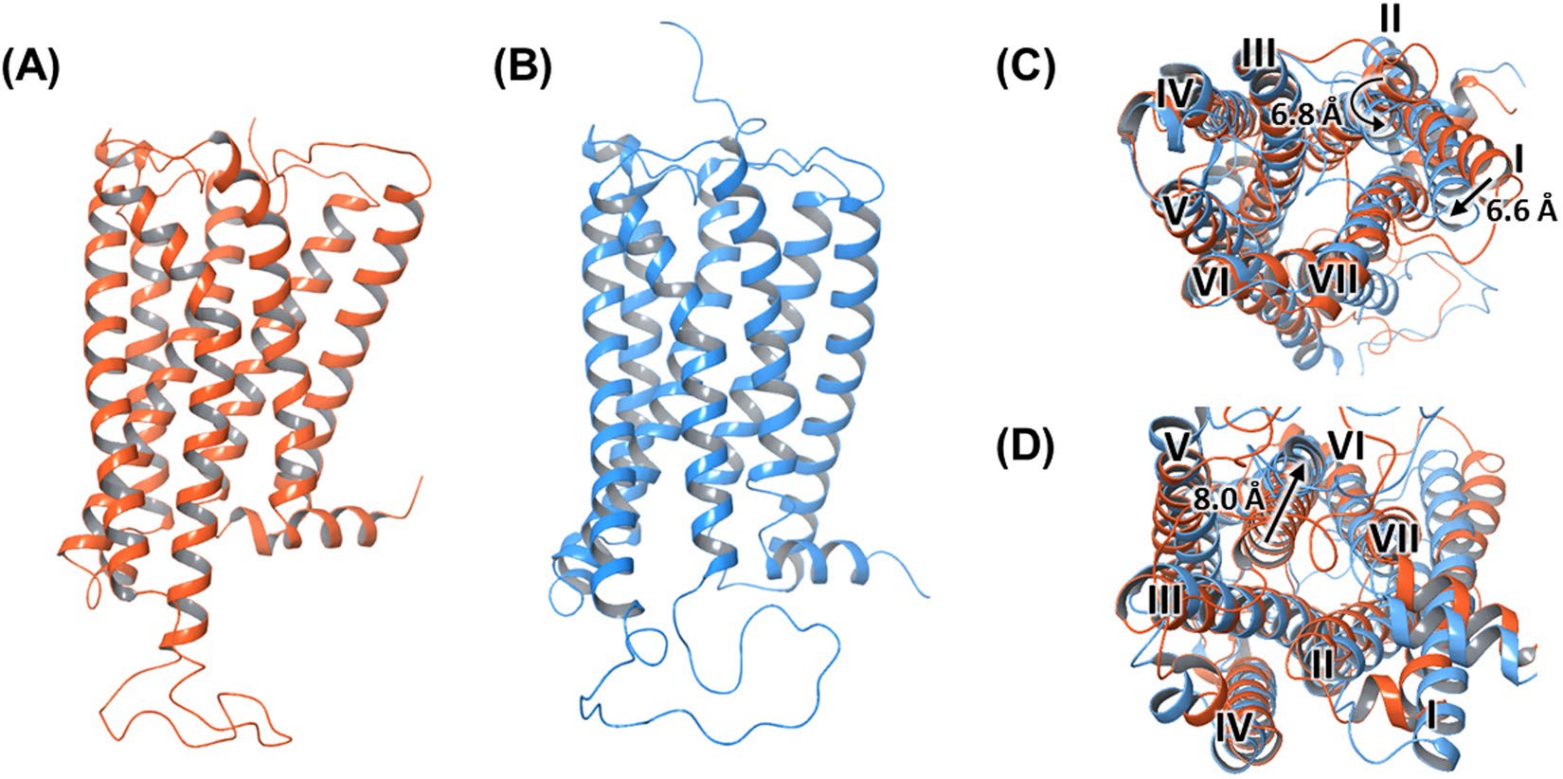
(**A**) Inactive conformation (**B**) active conformation, and the structural comparison of (**C**) extracellular and (**D**) intracellular part of two CB1 structures. To clarify the structural changes of two conformations of CB1, seven helices are labeled as I–VII.

Two intact CB1 structures, including the inactive and active conformations, were used for docking (Fig. 2A,B). The active conformation has notable structural changes as compared with the inactive conformation, especially in helices I, II, and VI^28^. The extracellular part of helix I and helix II were moved inwards by 6.6 Å and rotated inwards by about 6.8 Å, respectively, in the active conformation of the CB1 structure (Fig. 2C). In addition, the intracellular part of helix VI moved outwards by about 8 Å (Fig. 2D). Accordingly, the volume of the orthosteric ligand-binding site shrunk by 53% from 922 Å^3^ in the inactive conformation to 384 Å^3^ in the active conformation (Fig. S1). Therefore, it was expected that larger molecular weight ligands over 500 Da are hardly bound to the active conformation of the CB1 structure.

**Figure 3:**
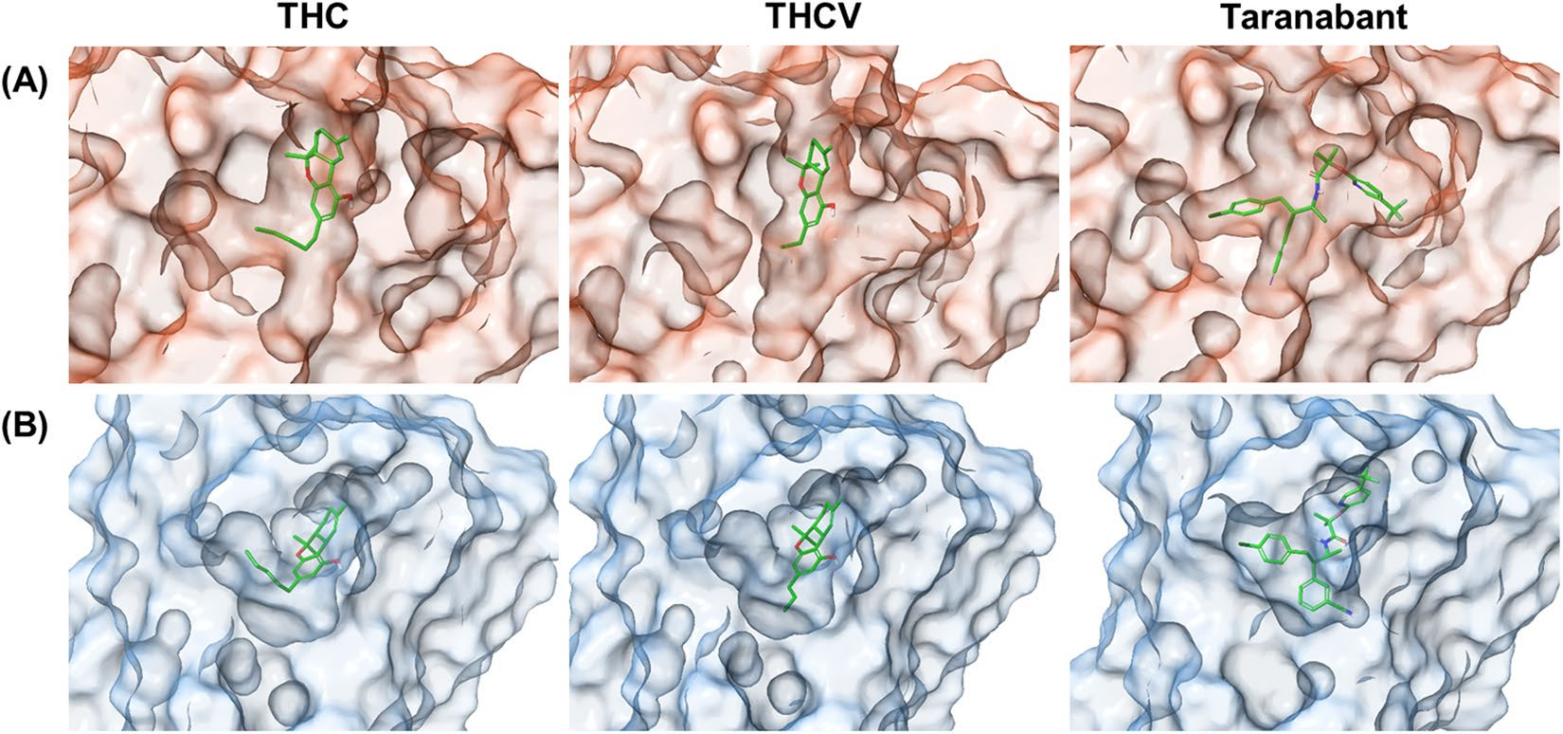
Binding poses of three ligands. The three ligands, including THC, THCV, and taranabant, were docked to the (**A**) inactive and (**B**) active conformations of CB1 receptor.

The three ligands were docked to the two conformations of CB1 by molecular docking for the prediction of the binding poses of ligands in the orthosteric ligand-binding site. The docking results are shown in Fig. 3. In case of THC and THCV, the ligands were docked well in both conformations and exhibited similar binding poses in the orthosteric ligand-binding site. The only difference between the two ligands was that the pentyl side chain of THC protrudes to the sub-pocket of the binding site, which was not shown in THCV. Taranabant was also docked well to the inactive conformation of CB1 but could not dock to the active conformation because of the large molecular weight (515.95 Da). To solve this problem, the induced-fit docking method was used to predict the possible binding poses of taranabant to the active conformation of CB1. The results showed that the binding pose of taranabant was different when comparing with the ligand docked to the inactive conformation, but its chemical groups were similarly positioned in sub-pockets. Consequently, six CB1-ligand complex structures were generated and were used for the following simulations.

### MD simulations of the apo and holo CB1 structures

**Figure 4:**
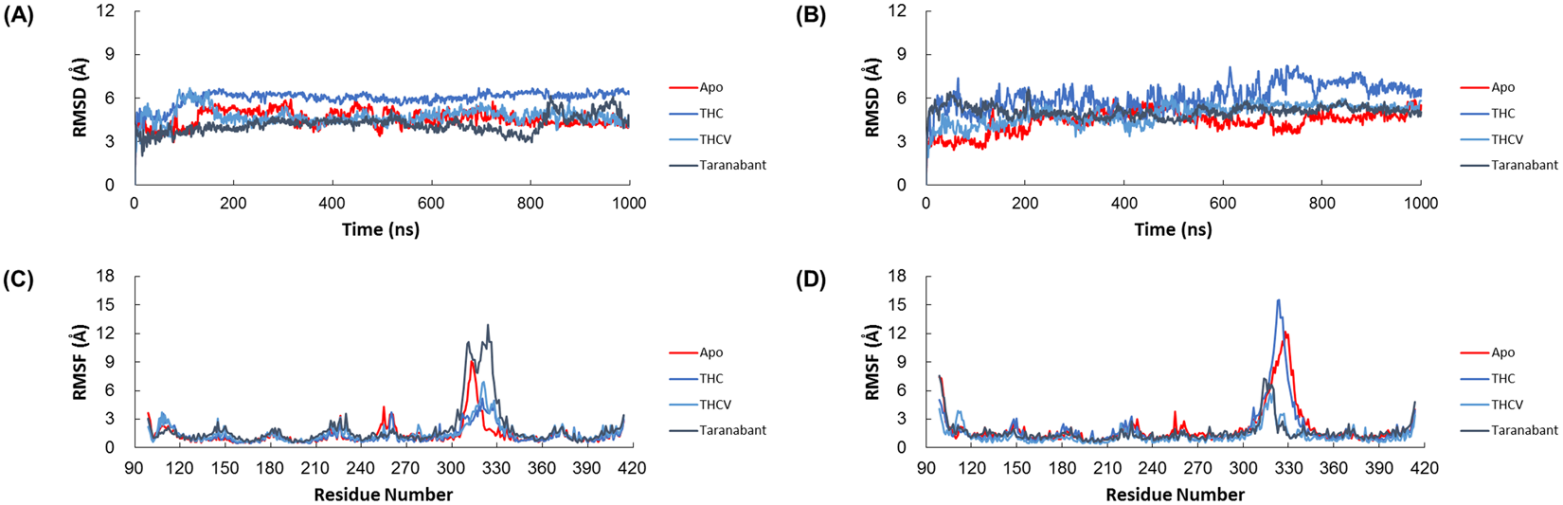
The RMSD and RMSF values obtained from MD simulations. The RSMD of the backbone atoms of (**A**) inactive and (**B**) active conformations of CB1 structures were calculated against their initial structures. The RMSF of the residues of (**C**) inactive and (**D**) active conformations of CB1 structures were also calculated against their initial structures.

MD simulations were carried out using two apo structures with different conformations and six holo structures generated by molecular docking. Next, the dynamic behaviors of eight structures were examined. In order to clarify the dynamic stability of these structures, RMSD values were obtained using the initial structures as templates (Figs 4A,B, and S2). The RMSD plot showed that the RMSD of the backbone atoms with respect to the initial structures increased for 200 ns. After that, they remained stable until the end of the simulation. Thus, the trajectories of the MD simulations of these structures were reliable.

RMSF values were then calculated to analyze the fluctuations of all residues (Fig. 4C,D). The extra- and intracellular loop regions of CB1 exhibited more fluctuations than helix regions. In both conformations of the CB1, the extra- and intracellular loop 3 (ECL3 and ICL3) fluctuated more than the other regions. It was demonstrated that the ECL3 and ICL3 regions was intrinsically flexible and had a potential to influence on neighbor helices.

### Analysis of the structural changes of CB1 upon ligand binding

**Figure 5:**
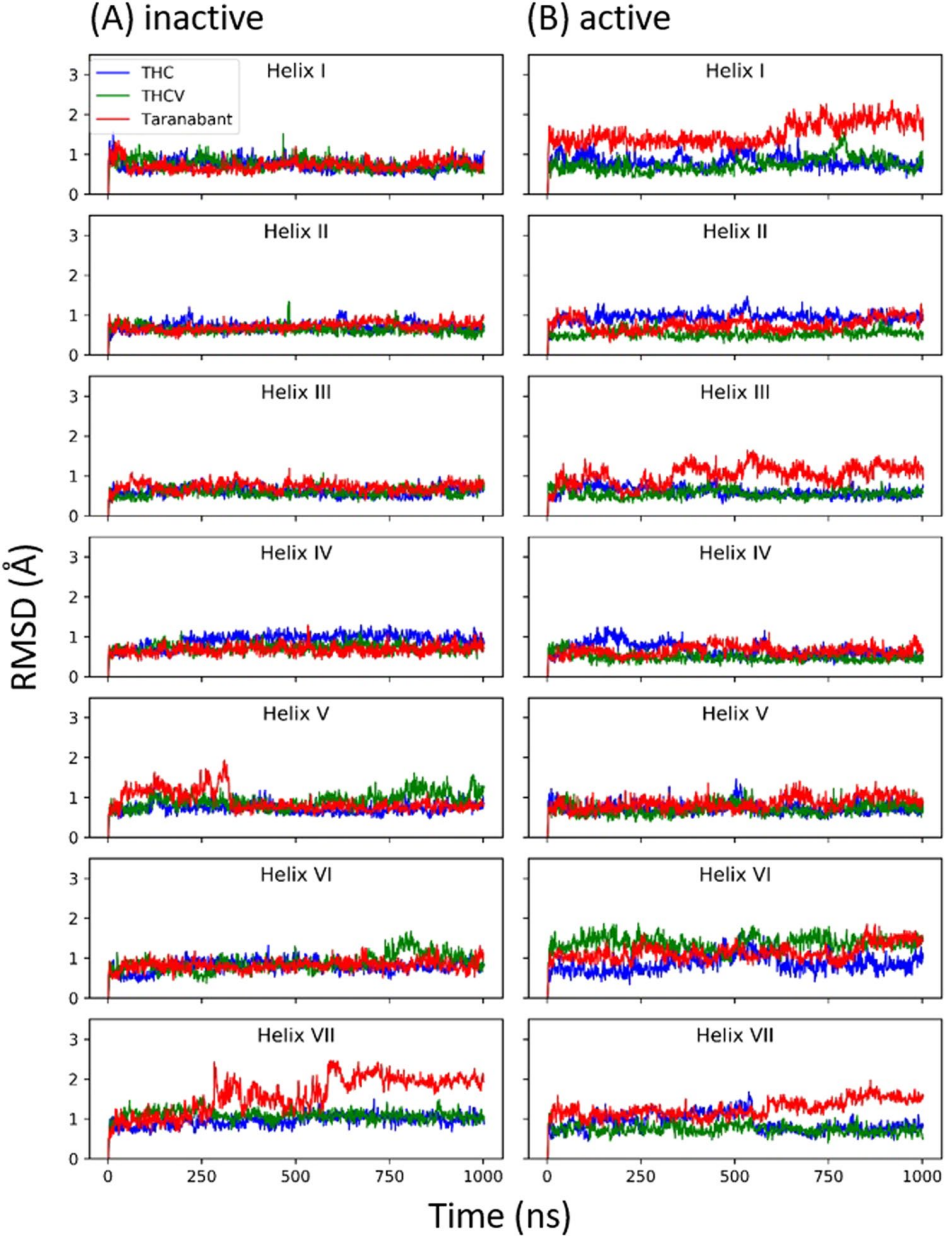
The RMSD values of each helix. The RSMD of each helix (helix I to helix VII) for (**A**) inactive and (**B**) active conformations of CB1 structures were calculated against their initial structures.

**Figure 6:**
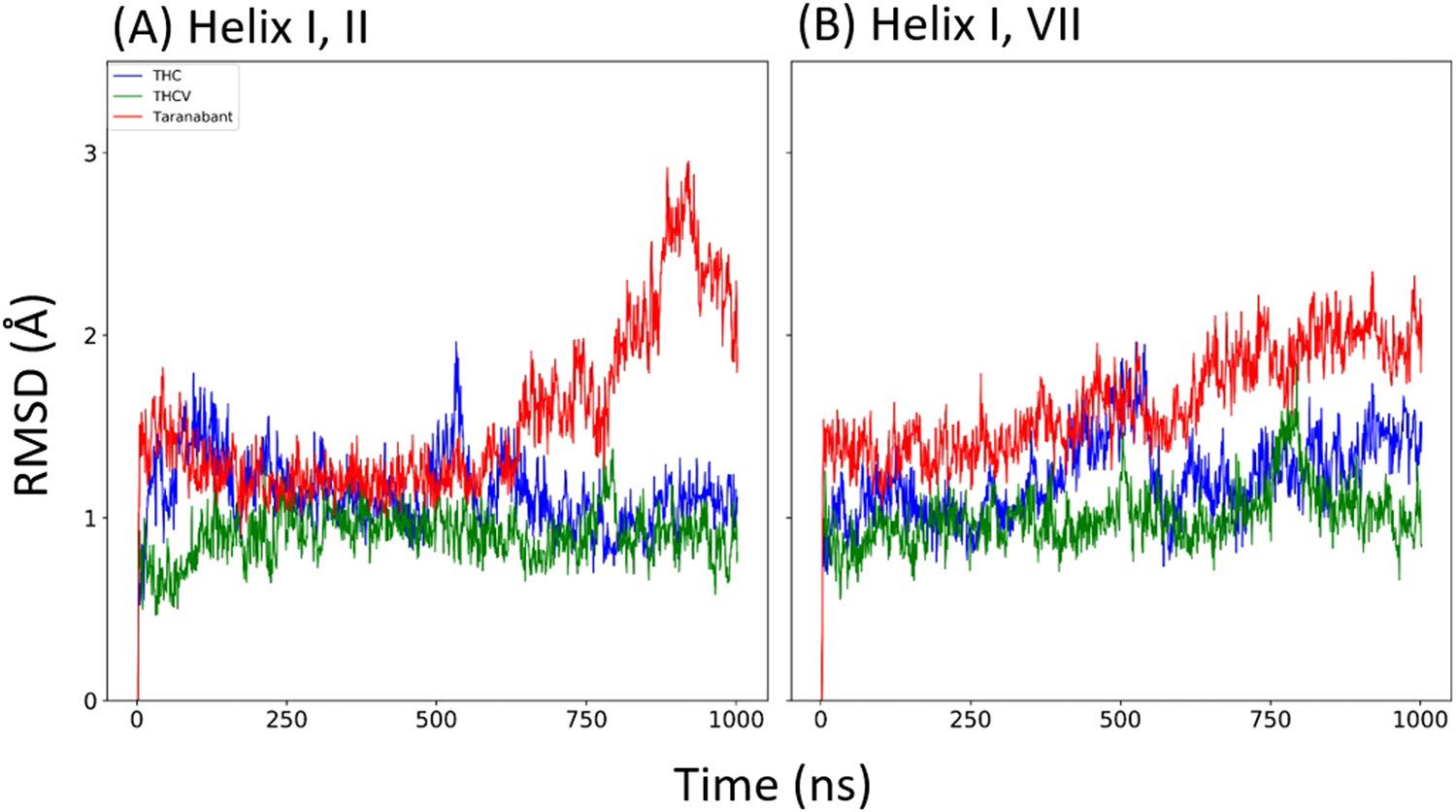
The RMSD values of helix pair in active state conformation. The RSMD of helix pairs (**A**) helix I, II and (**B**) helix I, VII.

In order to identify the dynamic behavior of seven transmembrane helices, RMSD value for each helix was calculated (Fig. 5). In addition, RMSD value for all possible contacts of helix pairs was calculated (Figs 6 and S3). When inverse agonist taranabant was bound to inactive conformation, helix VII was more dynamic than the partial agonist THC and the antagonist THCV were bound (Fig. 5A). However, RMSD of helix pairs showed similar RMSD values among the three ligands bound to inactive conformation (Fig. S3A). It was demonstrated that the dynamic behavior of helix VII did not affect the other helices. When taranabant was bound to the active conformation, helix I was more dynamic when comparing with the THC and THCV was bound (Fig. 5B). Moreover, the RMSD of helix I, II and helix I, VII pairs for taranabant bound to the active conformation was more dynamic than THC and THCV bound to the active conformation (Fig. 6). The RMSD of other helix pairs for taranabant bound to the active conformation was slightly more dynamic than the other ligands (Fig. S3B). The results showed that inverse agonist taranabant bound to the active conformation induced local conformational changes, which demonstrated the unfavorable interactions to the active conformation.

To identify the influences of the distinct structural changes of CB1 upon ligand binding, the residues around the orthosteric binding site were examined (Fig. S4). First, the orthosteric binding site of the two conformations was compared. A significant conformational change between Phe200 and Trp356 was identified, which was referred to as a twin toggle switch (Fig. S5)^28,71^. In the inactive conformation, Trp356 moved towards the orthosteric binding site and formed an aromatic stacking interaction with Phe200. However, in the active conformation, the cooperative rotation of helix III and the flipping of Phe200 allowed this residue to point towards the orthosteric binding site. At the same time, the outwards rotation of helix VI allowed Trp356 to move away from the orthosteric binding site, thereby disrupting the stacking interaction with Phe200. The different states of the twin toggle switch between the two conformations can influence the distinct structural changes of CB1 upon ligand binding. When three classes of ligands were bound to the inactive conformation, only taranabant stably interacted with Trp356. The *m*-CN benzyl group of taranabant contacted Trp356 thereby stabilizing the twin toggle switch during the simulations (Fig. S4A,B). These stable interactions were sufficient to maintain inactive conformation of CB1 as compared with other two classes of ligands which do not have chemical groups for binding. On the other hand, when three classes of ligands were bound to the active conformation, taranabant and THC stably interacted with Phe200. However, the twin toggle switch showed different distances (Fig. S4C,D). The distance between Phe200 and Trp356 was increased until 180 ns when THC was bound, while the distance was decreased when taranabant was bound. Then, the distance was reversed after 600 ns: Phe200 and Trp356 distance was decreased and stabilized when THC was bound, while the distance was increased when taranabant was bound. The movement of these two residues might influence the active conformation of CB1.

### Binding free energy analysis

In order to investigate the binding affinity of the three classes of ligands to the two conformations of CB1, the binding free energies were calculated using the MM/PBSA method (Tables 1 and 2). The binding free energies for the inactive conformation of CB1 with THC, THCV, and taranabant were 20.87, −21.02, and −41.49 kcal/mol, respectively. In addition, the binding free energies for the active conformation of CB1 with THC, THCV, and taranabant were −30.05, −28.03, and −31.30 kcal/mol, respectively. These binding free energies were significantly different according to the statistical analysis (SI Text and Fig. S6). THC and THCV were more favorably interacted with the active conformation than the inactive conformation. The binding energy of THC for the active conformation was higher than that of THCV, which was well related to inhibition constant (*K*_i_) values of the two ligands in several studies^21,72^. On the other hand, the inverse agonist taranabant was bound more favorably to the inactive conformation. According to the energy components of the binding free energies, the vdW term was the main driving force to ligand binding in both conformations. It implied that hydrophobic and aromatic residues are mainly located in the orthosteric binding site of CB1. The solvation energy term was unfavorable to ligand binding in both conformations. This term was increased depending on the size of the ligands. Thus, both terms should be considered first when designing a high affinity ligand for CB1.

**Table 1:** Binding free energy components for the inactive CB1-ligand complexes determined by using the MM/PBSA method.

Ligand	ΔE_MM_	ΔE_vdW_	ΔE_elec_	ΔE_polar_	ΔE_nonpolar_	ΔE_solv_	ΔE_bind_
THC	−43.24	−37.73	−5.51	10.99	11.38	22.37	−20.87
THCV	−54.65	−38.90	−15.75	23.86	9.77	33.63	−21.02
Taranabant	−82.64	−60.01	−22.63	25.83	15.32	41.15	−41.49

**Table 2:** Binding free energy components for the active CB1-ligand complexes determined by using the MM/PBSA method.

Ligand	ΔE_MM_	ΔE_vdW_	ΔE_elec_	ΔE_polar_	ΔE_nonpolar_	ΔE_solv_	ΔE_bind_
THC	−56.76	−52.83	−3.93	15.28	11.43	26.71	−30.05
THCV	−53.49	−42.87	−10.62	10.20	15.26	25.46	−28.03
Taranabant	−73.45	−59.47	−13.98	26.96	15.19	42.15	−31.30

**Figure 7:**
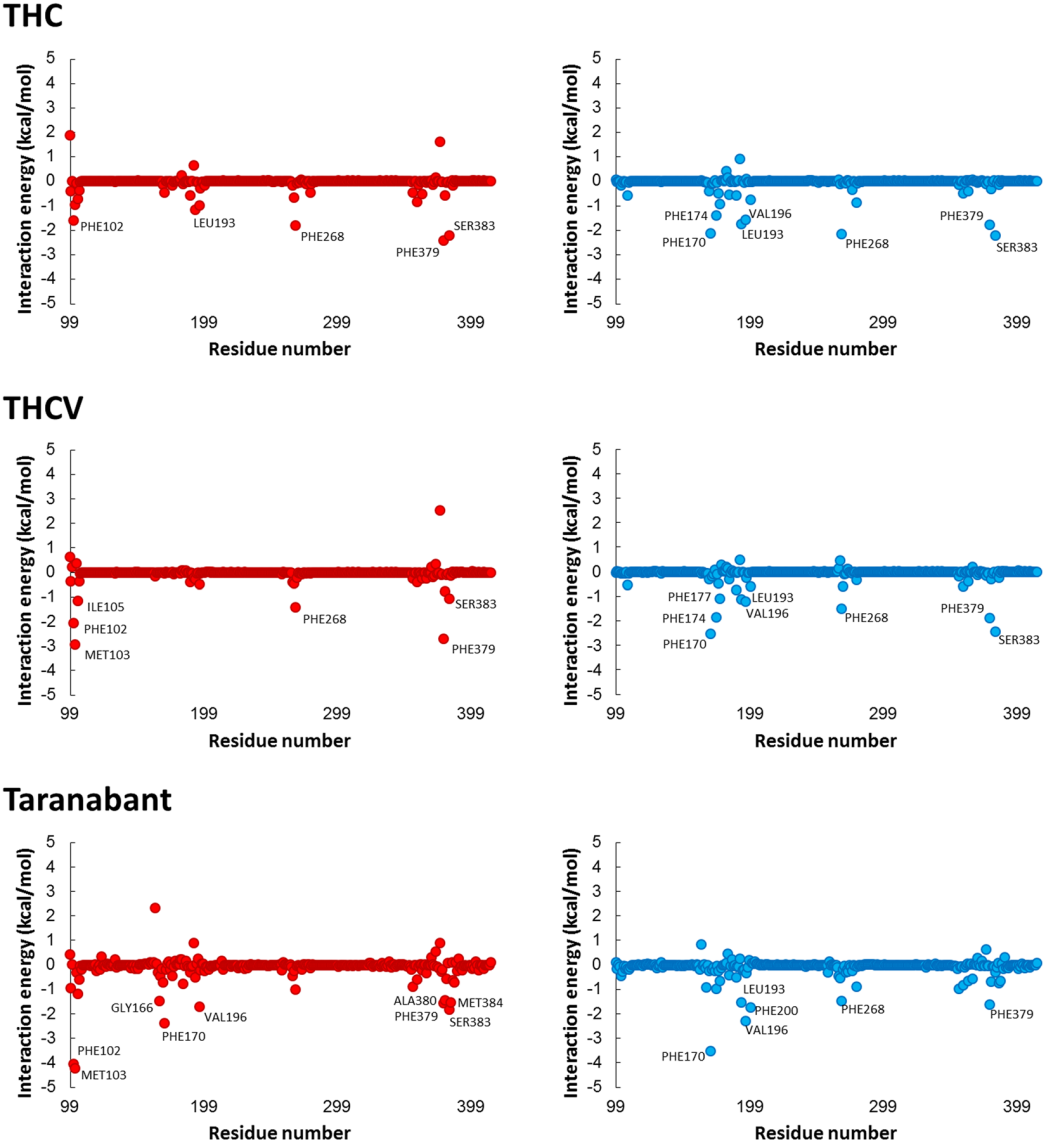
Per-residue binding free energy decomposition of inactive (left panel, red dots) and active (right panel, blue dots) CB1-ligand complexes. Residues with high energy contribution (the energy contribution ≤ −1.0 kcal/mol) were labeled.

**Figure 8:**
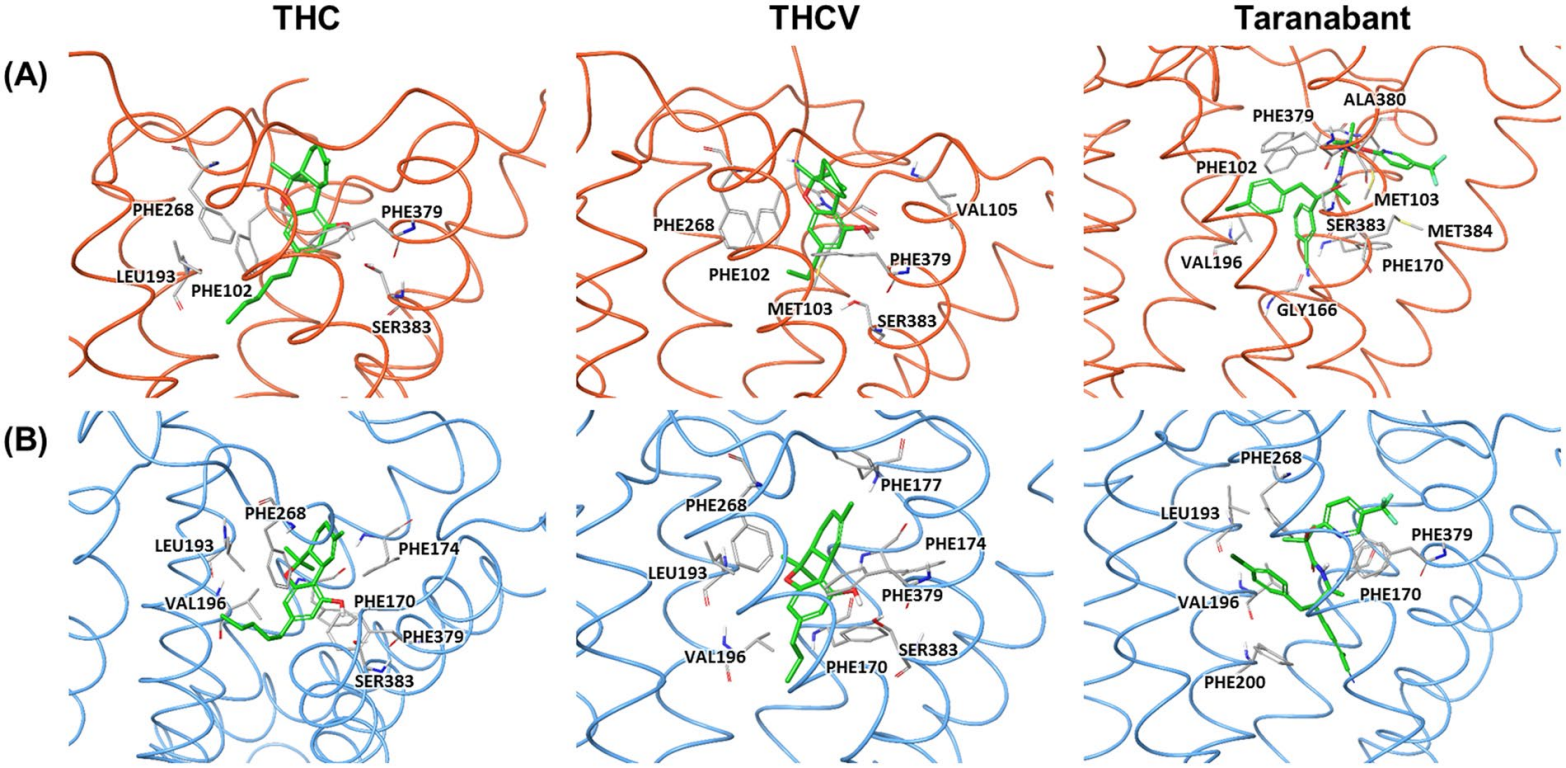
Binding poses of CB1 ligands after 1 μs MD simulations. Three ligands were bound to the (**A**) inactive and (**B**) active conformations of CB1 receptor. The residues with high energy contribution are displayed.

Next, we performed binding free energy decompositions of each residue in CB1 to identify the residues that are important for the interaction of the CB1-ligand complexes (Fig. 7). The binding poses of the three classes of ligands in the two conformations of CB1 and the residues with high energy contribution are displayed in Fig. 8. In the inactive conformation, three ligands were commonly interacted with three residues including Phe102, Phe379, Ser383. THC had an interaction with two additional residues, Leu193 and Phe268, while THCV interacted with three additional residues, Met103, Ile105, and Phe268. Although THCV interacted with more residues, THC was more strongly interacted with the residues inside the orthosteric binding site. Taranabant interacted with more residues located in the sub-pockets, the 2,4-dichlorophenyl ring interacted with one sub-pocket formed by Gly166 and Val196, and the piperidin-1-ylcarbamoyl part interacted with the other sub-pocket formed by Met103, Ala380, Ser383, and Met384. These additional interactions can strengthen the affinity of taranabant to the inactive conformation of CB1. In the active conformation, three ligands were commonly interacted with five residues including Phe170, Leu193, Val196, Phe268, and Phe379. THC and THCV interacted with similar residues, but THCV had an interaction with one more residue, Phe177. In this case, THC protruded the pnetyl side chain to the sub-pocket of the binding site, which induced tight binding of the ligand to inactive conformation than THCV. In case of taranabant, fewer residues were identified to interact when comparing with the ligand binding to the inactive conformation, thereby reducing the binding energy for the active conformation. Overall, binding free energy analysis demonstrated the discrimination of the three CB1 ligands. THC and THCV was favorably bound to the active conformation, whereas taranabant was favorably bound to the inactive conformation. Moreover, in case of THCV, the binding energy was smaller than other two ligands.

## Conclusion

In this study, we demonstrated the discrimination of the three classes of CB1 ligands, which have distinct efficacy profiles, using MD simulations and MM-PBSA method. The inactive and active conformations of wild-type intact CB1 structures were prepared first, and then the binding modes of three ligands were determined by molecular docking simulations. The results showed that THC and THCV were docked well in both conformations, while taranabant was docked only to the inactive conformation. It appears that there were binding preferences for the two conformations depending on the ligands.

The dynamics behaviors of helices and the twin toggle switches in orthosteric binding sites showed distinct structural profiles against the three classes of the ligands. In addition, binding free energies were calculated to investigate the binding affinity of the three classes of ligands to the two conformations of CB1 using the MM-PBSA method. THC and THCV were more favorably bound to the active conformation, while taranabant was favorably bound to the inactive conformation. In case of THCV, the binding energy was smaller than other two ligands. Thus, the different binding energies for the two conformations of CB1 can help to discriminate the ligand efficacy. All these observations demonstrated that the three classes of CB1 ligands can be discriminated in the two conformations of the intact CB1 structures by analyzing structural changes of both structures upon ligand binding. Moreover, binding free energy calculations can help to define the three classes of ligands. Our findings shed light on the understanding of different efficacy profiles of ligands by the structural behaviors of CB1 and the binding energies of ligands to yield insights useful for the design of new potent CB1 drugs.

## Electronic supplementary material

Supplementary information
