# Supplementary material for: Exploring the Ligand Efficacy of Cannabinoid Receptor 1 (CB1) using Molecular Dynamics Simulations
Source: Sci Rep. 2018 Sep 13;8:13787. doi: 10.1038/s41598-018-31749-z (PMC6137198; doi:10.1038/s41598-018-31749-z)
Supplement: Supplementary file 1 — Supplementary information [file 41598_2018_31749_MOESM1_ESM.docx]

**SUPPLEMENTARY INFORMATION**

**Exploring the Ligand Efficacy of Cannabinoid Receptor 1 (CB1) using Molecular Dynamics Simulations**

Sang Won Jung^1^, Art E. Cho^2*^, Wookyung Yu^1,3,4*^

^1^ *Center for Supercomputing and Big Data, Daegu Gyeongbuk Institute of Science and Technology (DGIST), 333 Techno jungang-daero, Daegu 42988, Korea*

^2^ *Department of Bioinformatics, Korea University, 2511 Sejong-ro, Sejong 30019, Korea*

^3^ *Department of Brain and Cognitive Sciences, Daegu Gyeongbuk Institute of Science and Technology (DGIST), 333 Techno jungang-daero, Daegu 42988, Korea*

^4^ *Core Protein Resources Center, Daegu Gyeongbuk Institute of Science and Technology (DGIST), 333 Techno jungang-daero, Daegu 42988, Korea*

* Corresponding Authors: [artcho@korea.ac.kr](mailto:artcho@korea.ac.kr) and [wkyu@dgist.ac.kr](mailto:wkyu@dgist.ac.kr)

**SI Text**

**Statistical analysis for binding free energy**

The distribution of binding free energy (Figure S6) shows the maximum peaks are separated and we can see the binding free energy difference. Also we performed statistical analysis of binding free energy, which determines the significance of binding free energy different between active conformations and inactive conformations. At first, we performed Shapiro-Wilk test whether binding free energy distributions are normal distribution or not. (1) The results of Shapiro-Wilk test show that binding free energy distribution of all conformations is normal distribution. (THC-active: W = 0.98921, p-value = 0.6013, THC-inactive: W = 0.97349, p-value = 0.04113, THCV-active: W = 0.98854, p-value = 0.5487, THCV-inactive: W = 0.9829, p-value = 0.222, Taranabant-active: W = 0.97498, p-value = 0.05381, Taranabant-inactive: W = 0.98162, p-value = 0.1775) Because all of the distributions are normal distribution, we performed t-test. T-test shows all binding free energy difference are significant: binding free energy of THC-active conformations is significantly lower than that of THC-inactive conformations, (t = -21.406, p-value < 2.2*10^-16^), binding free energy of THCV-active conformations binding free energy is significantly lower than that of THCV-inactive conformations, (t = -12.586, p-value < 2.2*10^-16^), binding free energy of Taranabant-inactive conformations binding free energy is significantly lower than that of Taranabant-active conformations, (t = 12.577, p-value < 2.2*10^-16^). We used R program for all statistical analysis.

**References**

(1) Shapiro, S. S. & Wilk, M. B. An analysis of variance test for normality (complete samples). *Biometrika*, **1965**, 52, 591–611.

**SI Figures**

**
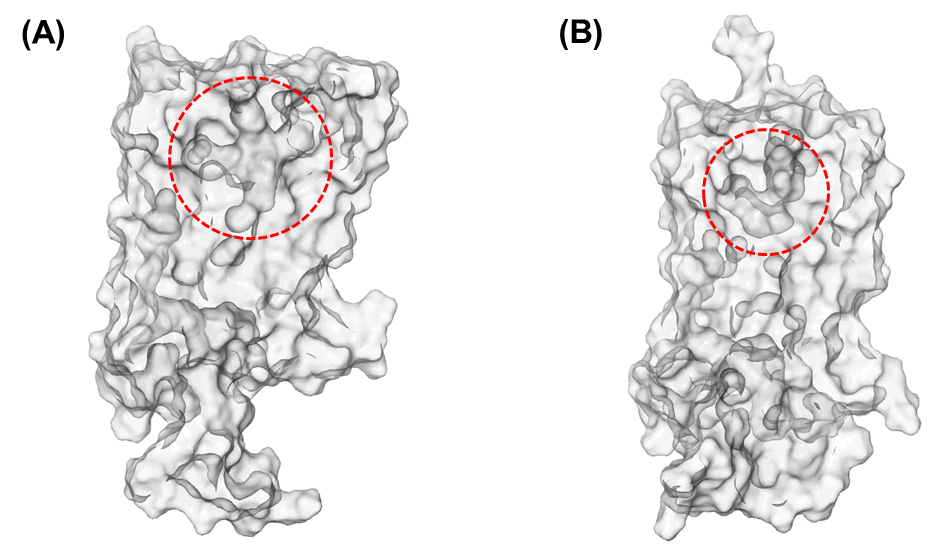
**

**Figure S1**. Comparisons of (A) inactive and (B) active conformations of CB1 orthosteric ligand-binding pockets. The orthosteric ligand-binding pockets are outlined by red dashed circles.

**
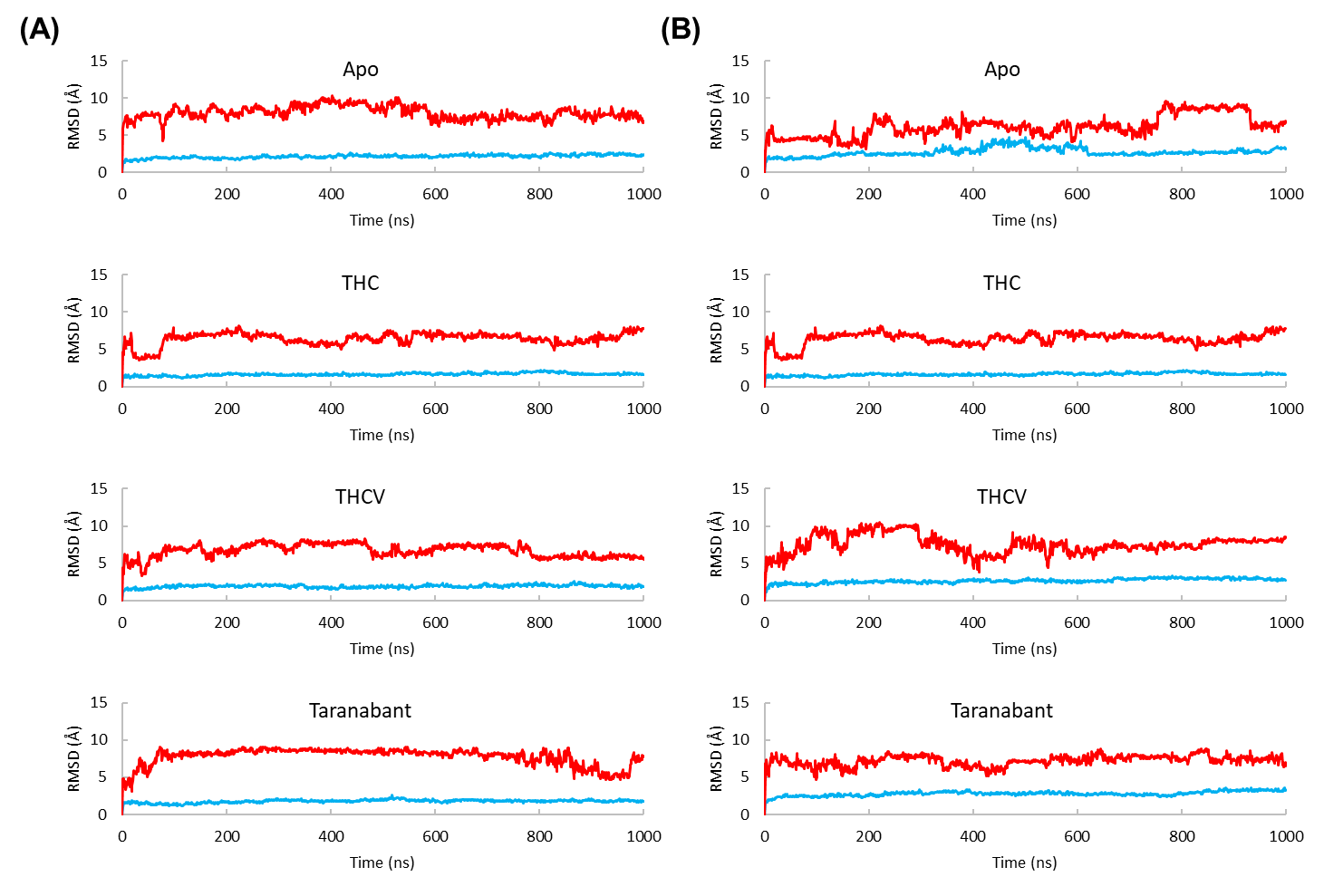
**

**Figure S2**. The RMSD of the backbone atoms of (A) inactive and (B) active conformations of CB1 structures against their initial structures. The ICL3 region is colored as red and the other regions are colored as blue.


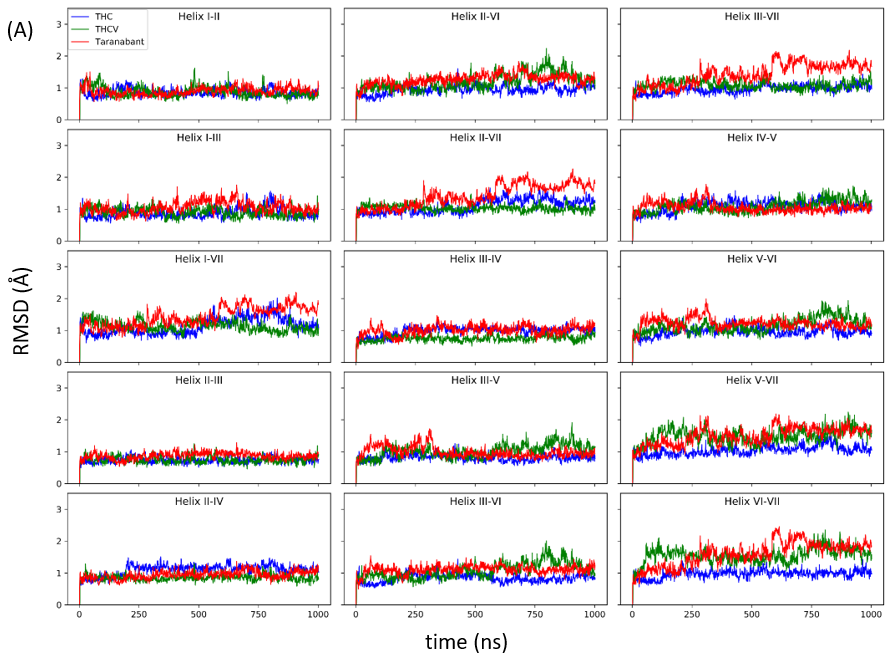


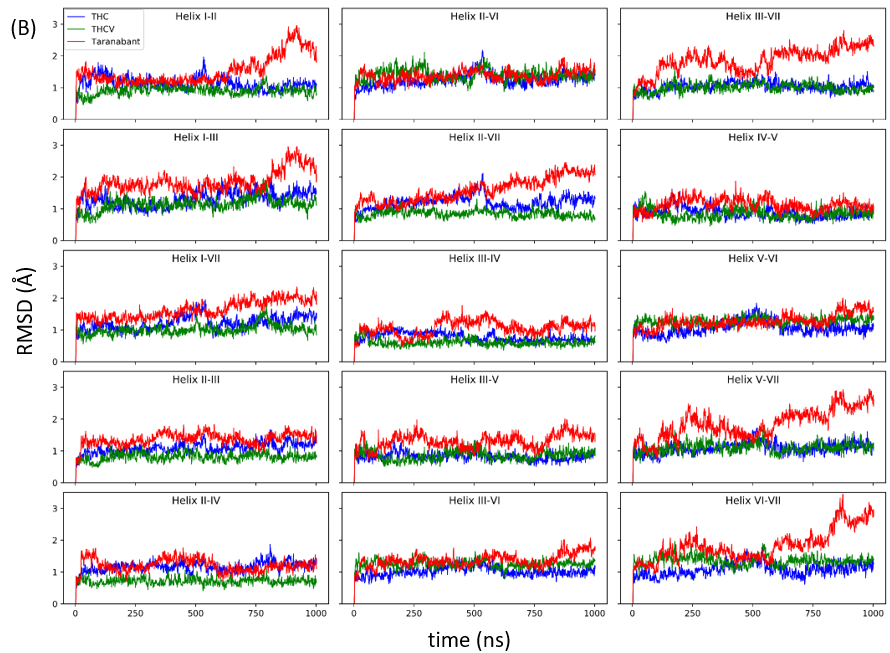


**Figure S3**. RMSD analysis for the contacts of helix pairs in (A) inactive and (B) active conformations


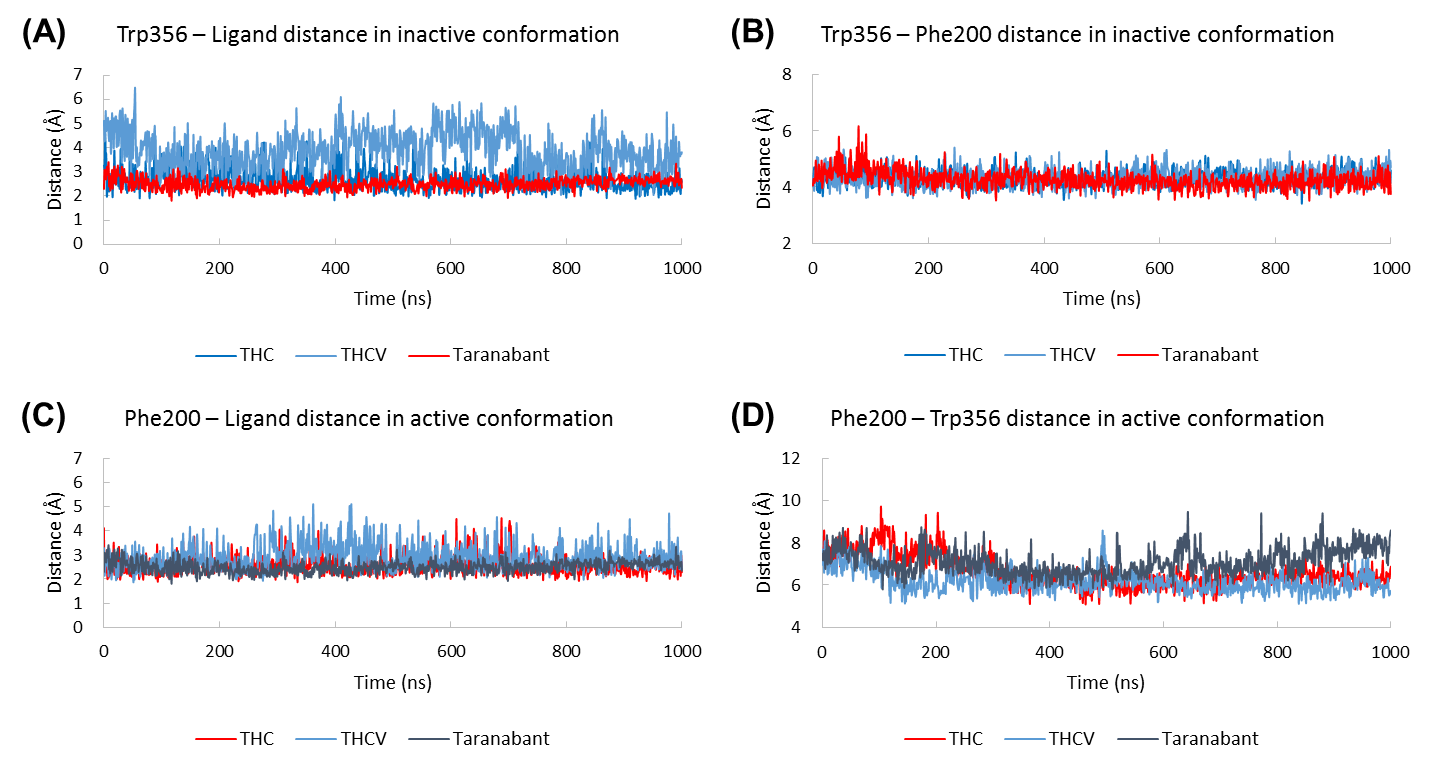


**Figure S4**. The time evolution of the distance between (A) Trp356-ligand and (B) Trp356-Phe200 in inactive conformation and (C) Phe200-ligand and (D) Phe200-Trp356 in active conformation.

**
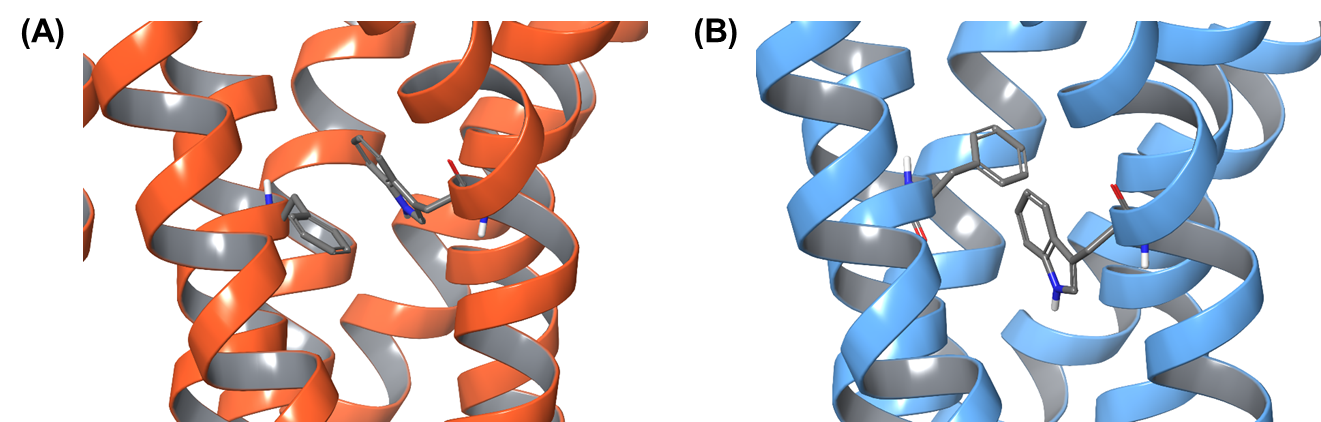
**

**Figure S5**. The structural comparisons of twin toggle switch, Phe200 and Trp356, in (A) inactive and (B) active conformation. Two residues are displayed in thick tube representation.


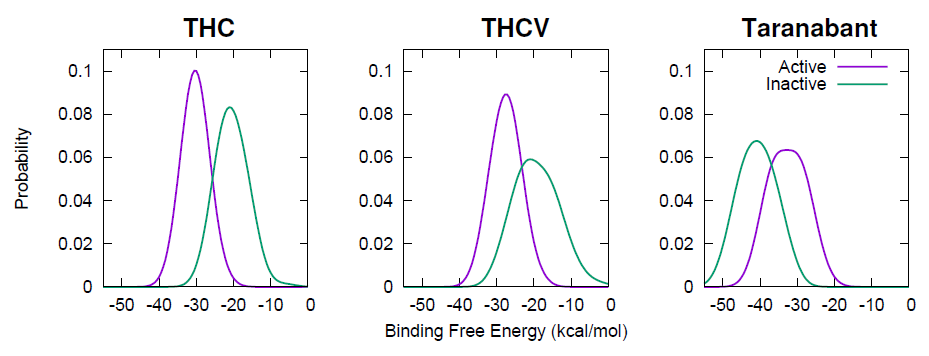


**Figure S6**. Binding free energy distributions of THC, THCV, and Taranabant for active (purple lines) and inactive (green lines) conformations of CB1.
